# Regional Diversities in Fibrogenesis Weighed as a Key Determinant for Atrial Arrhythmogenesis

**DOI:** 10.3390/biomedicines9121900

**Published:** 2021-12-14

**Authors:** Cheng-Chih Chung, Chye-Gen Chin, Yung-Kuo Lin, Yao-Chang Chen, Wan-Li Cheng, Yung-Hsin Yeh, Yu-Hsun Kao, Yi-Jen Chen

**Affiliations:** 1 Division of Cardiology, Department of Internal Medicine, School of Medicine, College of Medicine, Taipei Medical University, Taipei 11031, Taiwan; michaelchung110@gmail.com (C.-C.C.); yklin213@yahoo.com.tw (Y.-K.L.); 2 Division of Cardiovascular Medicine, Department of Internal Medicine, Wan Fang Hospital, Taipei Medical University, Taipei 11696, Taiwan; gen2892666@hotmail.com; 3 Taipei Heart Institute, Taipei Medical University, Taipei 11031, Taiwan; 4 Graduate Institute of Clinical Medicine, College of Medicine, Taipei Medical University, Taipei 11031, Taiwan; 5 National Defense Medical Center, Department of Biomedical Engineering, Taipei 11490, Taiwan; yaochang.chen@gmail.com; 6 Division of Cardiovascular Surgery, Department of Surgery, Wan Fang Hospital, Taipei Medical University, Taipei 11696, Taiwan; wanlicheng80@gmail.com; 7 Division of Cardiovascular Surgery, Department of Surgery, School of Medicine, College of Medicine, Taipei Medical University, Taipei 11031, Taiwan; 8 Division of Cardiology, Chang Gung Memorial Hospital, Taoyuan 33305, Taiwan; yeongshinn@cgmh.org.tw; 9 School of Medicine, College of Medicine, Chang Gung University, Taoyuan 33302, Taiwan; 10 Department of Medical Education and Research, Wan Fang Hospital, Taipei Medical University, Taipei 11696, Taiwan

**Keywords:** fibroblasts, fibrosis, atrial fibrillation, heart failure, left atrium, right atrium, Ca^2+^, transforming growth factor, oxidative stress, nitric oxide

## Abstract

Atrial fibrosis plays a key role in atrial myopathy, resulting in the genesis of atrial fibrillation (AF). The abnormal distribution of fibrotic tissue, electrical coupling, paracrine interactions, and biomechanical–electrical interactions have all been suggested as causes of fibrosis-related arrhythmogenesis. Moreover, the regional difference in fibrogenesis, specifically the left atrium (LA) exhibiting a higher arrhythmogenesis and level of fibrosis than the right atrium (RA) in AF, is a key contributor to atrial arrhythmogenesis. LA fibroblasts have greater profibrotic cellular activities than RA fibroblasts, but knowledge about the regional diversity of atrial regional fibrogenesis remains limited. This article provides a comprehensive review of research findings on the association between fibrogenesis and arrhythmogenesis from laboratory to clinical evidence and updates the current understanding of the potential mechanism underlying the difference in fibrogenesis between the LA and RA.

## 1. Introduction

Atrial fibrosis is a distinctive pathological finding of atrial myopathy and contributes to the genesis of various cardiovascular diseases. A higher level of left atrium (LA) fibrosis is associated with a greater recurrence of atrial fibrillation (AF) after surgical management or catheter ablation [1,2]. Patients with heart failure (HF) exhibited a higher prevalence of atrial fibrosis [3], and mineralocorticoid receptor antagonist treatment decreases the incidence of new-onset AF in patients with HF [4]. AF also increases the area of fibrosis in patients with HF [5], and the crosstalk between fibrogenesis and arrhythmogenesis thus plays a pivotal role in AF. For decades, numerous studies about antiatrial remodeling therapy, also called “upstream therapy”, for AF management have focused on this crucial process. The LA exhibits greater levels of fibrosis compared with the right atrium (RA) in patients with AF, according to the results of fibrotic tissue staining [6,7,8]. In addition, the LA exhibits higher gene expression of collagen I and collagen III compared to the RA [8]. Moreover, LA biopsies exhibit greater expression of pro-fibrotic transforming growth factor (TGF)-β and angiotensin (Ang) II, compared with RA biopsies [8]. By contrast, immunohistological staining reveals higher collagen I and III expression in RA tissue than in LA tissue [9]. Studies of LA and RA regional biopsies have reported heterogenous conclusions about the atrial regional fibrotic diversity, which may be because of the differences between studies in the area being sampled. Studies have also demonstrated that late-gadolinium enhancement magnetic resonance imaging (LGE-MRI) can detect the fibrosis of the whole LA or RA [10]. Notably, in patients with AF, the majority of the LGE in the RA is located in the intra-atrial septal region with lower enhancement in the remainder of the RA. The levels of LGE are considerably lower in the whole RA than in the LA, indicating that the RA has a lower level of fibrosis than the LA [11]. In a study using a sheep model, the activation frequencies in certain areas of the LA were always faster than those in the RA [12,13]. Further, results from the noncontact mapping of biatrial activation in canines with HF have demonstrated that the LA has a higher frequency of focal discharge and greater effective refractory period dispersion than the RA [14]. Four possible interactions between fibroblasts and cardiomyocytes may result in atrial arrhythmogenesis: (1) abnormal distribution of fibrotic tissue, (2) modulations in electrical coupling, (3) paracrine interactions, and (4) biomechanical–electrical interactions [15,16]. Moreover, the LA and RA exhibit diverse characteristics in cytokine production [17], gene expression [18,19], and epigenetic modification [20], which may contribute to their different fibrogenesis. This review clarifies the crosstalk mechanisms of fibrogenesis and arrhythmogenesis and the regional differences associated with the diversity of fibrogenesis between the LA and RA. This review also provides an update on the existing body of laboratory and clinical evidence, for fibrosis-related electrophysiological diversity between the LA and RA. 

### 1.1. Abnormal Distribution of Fibrotic Tissue in Atrial Electrical Coupling and Arrhythmogenesis

Atrial fibrotic tissue induces a unidirectional block, leading to the development of reentry [21]. Impulse conduction is forced to travel through the tortuous electrically insulating conduit formed by the fibrotic tissue, thereby inducing slow, discontinuous, or fractionated electrical wave propagation, inducing reentry [22,23]. Propagation waves perpendicular to the collagen fiber exhibit slower conduction velocity [23]; hence, the organization of fibrotic tissue may be highly correlated with reentry stability. Atrial fibrosis can be classified as patchy and diffuse type, according to its architecture [24]. Patchy fibrosis contributes to wavebreak, slower conduction, and a higher incidence of reentry multiplication rather than diffuse fibrosis [24]. In normal myocardium, connexins are located primarily at the end of intercalated disks, which contribute to the impulse conduction of myocyte–myocyte coupling. AF with atrial fibrosis exhibits the heterogenous redistribution of Cx43 in the atrial myocardium [25]. The lateralization of gap junctions induces lower conduction velocity in AF with atrial fibrosis [26]. Besides, atrial myofibroblasts isolated from the surgical specimens of the patients with AF exhibit high Cx43 expression [27]. This finding indicates that fibrogenesis may induce the remolding of the gap junction and activate arrhythmogenesis.

Studies have revealed that atrial myofibroblasts maintain their resting membrane potential of approximately −30 mV, which is considerably less negative than that of myocytes [27]. Myocytes, when coupled with an increased number of myofibroblasts, exhibit greater depolarized resting membrane potential, thereby inactivating sodium channels, decreasing conduction velocity, and increasing the complexity of wave propagation [28]. Accordingly, the distribution and density of atrial myofibroblasts–cardiomyocytes heterocellular coupling may be a key element of atrial arrhythmogenesis.

### 1.2. Fibrosis-Related Impairment of Biomechanical–Electrical Properties

The resting membrane potential of atrial fibroblasts can be modulated by cardiac contraction stretch through mechanosensitive channels, thereby depolarizing the coupled cardiomyocytes and slowing down conduction velocities [29]. A simulation study revealed that atrial fibroblasts–myocytes coupling through the stretch-activated ion channel prolonged repolarization, action potential duration, and depolarized the resting potential of human atrial myocyte, thereby slowing down wave propagation and decreasing strain in fibrotic tissue [30]. Patients with AF exhibited higher levels of atrial stiffness compared with patients with sinus rhythm because of atrial fibrosis [31], suggesting that the higher levels of atrial fibrosis contribute to increased heterogenous impulse wave transmission and conduction block.

### 1.3. Enhancement of Atrial Arrhythmogenesis by Paracrine Signaling from Fibrosis

Paracrine mediators contribute to the indirect communication between cardiomyocytes and myofibroblasts. Atrial myofibroblast-secreted platelet-derived growth factor induces calcium channel remodeling and shortening of action potential duration of coupled atrial myocytes [32]. TGF-β1 increases ion-channel remodeling of atrial myocytes [33]. Besides, transgenic mice with TGF-β1 overexpression exhibit atrial heterogenous conduction and prolonged intracellular calcium transient with selective atrial fibrosis, leading to high AF vulnerability [34,35]. Our previous study verified that LA fibroblasts secreted greater TGF- β1 than RA fibroblasts [36]; hence, the paracrine interaction of TGF-β1 may explain the diversity in arrhythmogenesis between the LA and RA. MicroRNA(Mir) is a small noncoding RNA of approximately 22 nucleotides that can negatively modulate gene expression [37] through mRNA degradation, translation inhibition, or transcriptional inhibition [38,39]. Atrial myofibroblasts-derived exosomes decrease the Cav1.2 gene expression of cardiomyocytes through Mir-21-3p, thereby decreasing the L-type Ca^2+^ current and increasing AF vulnerability [40,41]. Oxidative stress is a central mediator of AF and atrial fibrosis [42,43]. Notably, in our previous study, we observed that LA fibroblasts produce higher oxidative stress than RA fibroblasts [36]. Moreover, oxidative stress increases intracellular Ca^2+^ overloading, thereby inducing delayed afterdepolarization of atrial myocytes [44]. Therefore, oxidative stress may not only contribute to the dissimilarities of atrial fibrogenesis but also augment the diversity of atrial arrhythmogenesis through fibrogenesis. Consequently, the myofibroblast may not only play a passive role as an impulse conduction conduit but also be an active modulator of arrhythmogenesis through paracrine interaction. Moreover, cultured atrial myocytes after rapid pacing (10 Hz) can also increase collagen and TGF- β1 production of atrial fibroblasts through the secretion of Ang II [45]. Besides, atrial myocytes were found to produce calcitonin, acting as a paracrine signal that inhibits migration, proliferation, and collagen production of neighboring atrial fibroblasts [46]. These findings suggest that paracrine factors from the atrial myocytes may also affect the development of fibrosis.

## 2. Diversity in Atrial Fibrogenesis and Arrhythmogenesis: Clinical Evidence

Electroanatomic mapping results have demonstrated that the average conduction velocities are slower in the LA than in the RA in patients with AF [47], suggesting a key role for regional differences in fibrosis in the pathophysiology of AF. The average cycle length in LA is shorter than that in the RA in AF patients [48], indicating that the LA has greater reentrant activation than the RA. Sites that exhibited high-frequency activity compared with the surrounding atrial tissue are defined as dominant-frequency (DF) sites, which can also be identified as the atrial tissue sustaining rotational activity in fibrillation [49]. DF mapping is used to identify the localized sites of maximal DF during AF [50]. LA DFs are considerably greater than RA DFs in AF patients [7]. Interestingly, collagen I deposition in atrial tissue is highly positively correlated with the levels of DF in patients with AF [7], which provides strong evidence to support the crosstalk between atrial arrhythmogenesis and fibrogenesis. The rotor frequency identified by optical mapping was found to correlate well with the DF, as determined by signal analysis [51]. Rotors, representing stable but meandering spiral waves, can anchor to areas of anatomic discontinuity such as fibrosis [52], and therefore, voltage mapping may reveal sites important to maintaining atrial rotational activity. In patients with AF, the AF rotors are primarily located in the heterogenous LGE areas. Moreover, the combination of LGE-MRI and 3D phase-mapping makes it feasible to precisely identify the fibrogenic and arrhythmogenic substrates correlated with the AF rotor [53]. A previous study using a mapping strategy, which targeted the focal impulse and rotor modulation with a novel 64-electrode basket catheter in AF patients, revealed that the location of rotors is higher in the LA than in the RA, with a ratio of 4.54:1 [54]. This is highly correlated with the LGE-MRI fibrosis imaging, which demonstrates that the LA features greater enhancement relative to the RA [11]. Accordingly, these clinical images and novel diagnostic tools indicated the atrial regional diversities in electrical and structural remodeling, which may provide further information for planning electrical physiological ablation or drug discovery.

### Laboratory Evidence of Diversity in Atrial Fibrogenesis and Arrhythmogenesis

**Figure 1:**
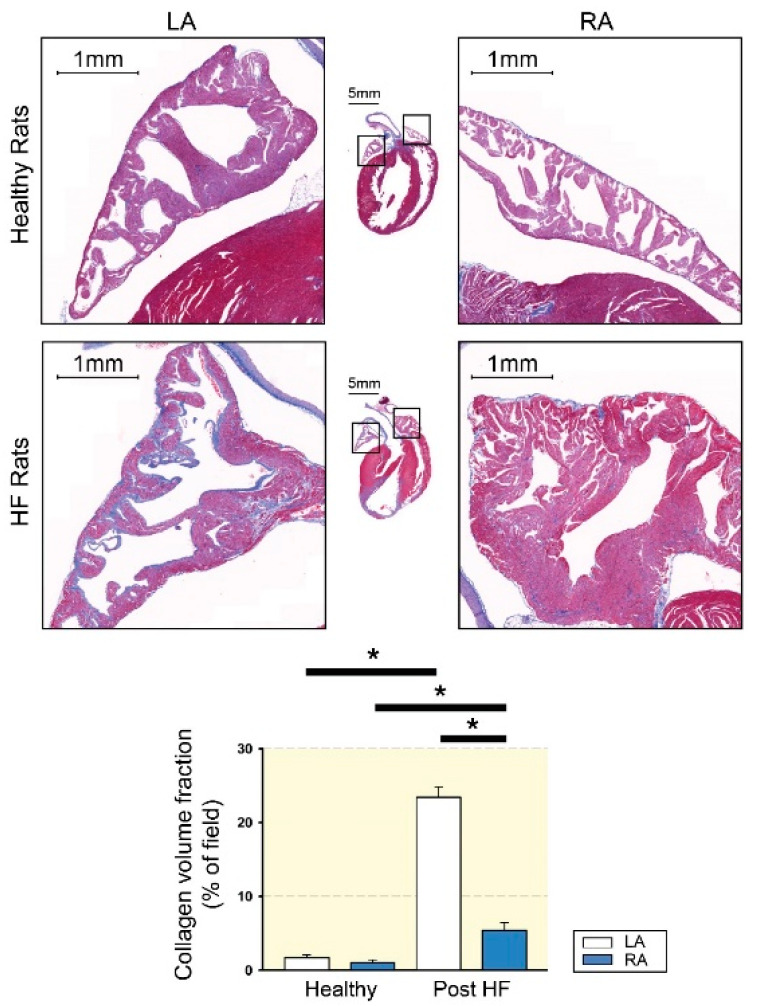
Atrial fibrosis of left atrium (LA) and right atrium (RA) tissues from healthy and heart failure with reduced ejection fraction (HF) rats. The upper photographs show representative photos with Masson’s trichrome staining of healthy LA and healthy RA atrial fibrosis. The lower photographs show representative photos of HF LA and HF RA atrial fibrosis. Compared to HF RA, HF LA exhibited greater levels of atrial fibrosis. LA and RA from healthy rats revealed a similar extent of atrial fibrosis. HF increased fibrosis to a greater severity in LA than in RA. * *p* < 0.05 (modified from [36] with permission of the publisher).

Similar to the results of the human study [48], those of our previous study revealed that LA tissue exhibits higher atrial fibrosis than RA tissue in rats with HF with reduced ejection fraction assessed by echocardiography (Figure 1) [36]. Similarly, in dogs with atrial myopathy, the mean atrial fibrillatory cycle length is shorter in the LA than in the RA, suggesting that the LA has higher fibrogenesis [55]. In addition, a sheep AF model revealed the left-to-right decrease in DF [56], suggesting that LA may be the source of higher-frequency activation, which transmits fibrillatory conduction to RA, thereby inducing AF. Moreover, in mitral regurgitation pigs, which are highly vulnerable to AF, the area of fibrosis in the LA is larger than that in the RA, but the conduction velocity of the LA is lower than that of the RA [57]. This study also found that conduction velocity is negatively correlated with the area of fibrosis [57]. These pieces of laboratory evidence confirm the regional heterogenicity and the possible crosstalk between fibrogenesis and arrhythmogenesis.

## 3. Mechanisms Contributing to the Regional Diversities of Atrial Fibrogenesis

Ali et al. used lineage tracing and histochemistry to study the origins of cardiac fibroblasts in transgenic mice and found that the majority of the neural crest-derived fibroblasts were localized in RA [58]. This finding suggests that part of the RA fibroblasts may come from different embryonic germ layers to those of LA fibroblasts. In addition, the dissimilar characteristics between LA and RA fibroblasts are correlated with the diversity in oxidative stress, nitric oxide (NO) signaling, TGF-β production, and Ca^2+^ signaling [36,59,60]. Table 1 summarizes the potential targets underlying different fibrogenesis between the RA and LA.

### 3.1. NO and Ca^2+^ Signaling Modulation

NO plays a pivotal role in fibrogenesis and AF. NO is a highly reactive radical that is generated from L-arginine by NO synthase (NOS). Inhibition of NOS can enhance myocardial fibrosis [96]. The NO/soluble guanylyl cyclase (sGC)/cyclic GMP (cGMP) signal pathway can decrease Ang II-induced profibrotic mitogen-activated protein kinase activities or attenuate collagen production of fibroblasts by interfering with phosphorylated SMA and MAD-related protein (Smad) 2 expression [97,98]. Endothelial NOS (eNOS) gene expression negatively correlates with the risk of AF [99]. Compared with LA tissue, RA tissue exhibits higher NO, eNOS, and sGC expression [6,61], indicating that the RA produces greater levels of NO, thereby decreasing the cellular activities of RA fibroblasts and attenuating RA fibrogenesis. Moreover, in different mouse strains and human atrial tissue experiments, the RA exhibited greater gene expression of adrenomedullin than the LA [18], indicating that this endothelial cell and vascular smooth muscle cell-secreted peptide can contribute to atrial regional diversity in fibrogenesis caused by the dissimilarity in NO/sGC signaling [100]. 

**Figure 2:**
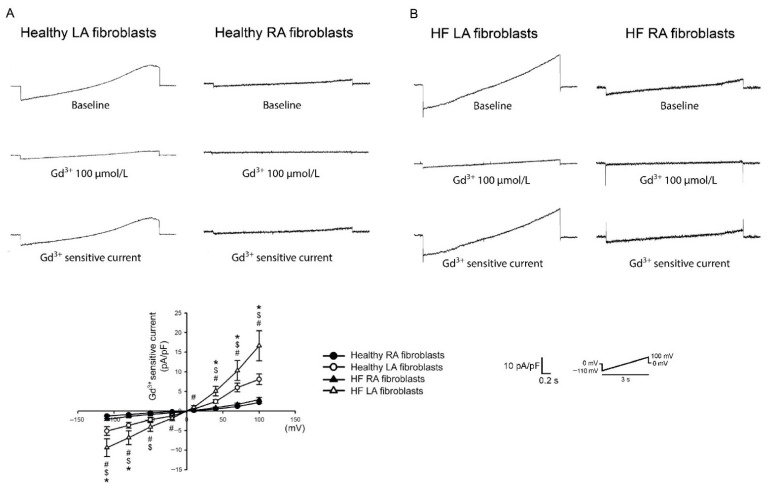
Membrane gadolinium (Gd^3+^)-sensitive Ca^2+^ currents of the left atrium (LA) and right atrium (RA) fibroblasts in healthy and heart failure with reduced ejection fraction (HF) rats. (**A**). Left and right panels, respectively, reveal tracings of the Gd^3+^ (100 μmol/L)-sensitive nonselective cation current of LA and RA fibroblasts isolated from healthy rats. Healthy LA fibroblasts exhibited greater Gd^3+^-sensitive Ca^2+^ currents compared with healthy RA fibroblasts. (**B**). Left and right panels, respectively, reveal tracings of the Gd^3+^ (100 μmol/L)-sensitive nonselective cation current of LA and RA fibroblasts isolated from HF rats. The statistical results revealed that healthy LA fibroblasts exhibited greater Gd^3+^-sensitive Ca^2+^ currents compared with healthy RA fibroblasts. HF LA fibroblasts exhibited greater Gd^3+^-sensitive Ca^2+^ currents compared with HF RA fibroblasts. LA fibroblasts from HF rats showed higher Gd^3+^-sensitive currents compared with LA fibroblasts from healthy rats. The insets in the current traces showed the various clamp protocols. * Healthy LA versus healthy RA fibroblasts; $ HF LA versus healthy LA fibroblasts; # HF LA versus HF RA fibroblasts (adapted from the published article by Chung et al. [59]).

Ca^2+^ homeostasis plays a key role in the pathophysiology of cardiac fibrosis. Ca^2+^ signaling augments the profibrotic cellular activities of fibroblasts [101,102,103,104]. Extracellular Ca^2+^ entry and endoplasmic reticulum (ER) Ca^2+^ release contribute to the increase in intracellular Ca^2+^. In our previous study, we found that, compared with RA fibroblasts, LA fibroblasts exhibited higher Ca^2+^ entry, thereby inducing greater collagen production (Figure 2) [59]. In addition, compared with RA tissue, LA tissue exhibited greater chymase production, which is a profibrotic protease that can induce Ca^2+^ influx [85,105,106]. The cardiovascular neurotransmitter calcitonin gene-related peptide, highly expressed in LA tissues but not in RA tissue, can increase the amount of intracellular Ca^2+^ through Ca^2+^ entry [86,107]. Moreover, compared to LA tissue, RA tissue exhibits higher estrogen receptor-α expression, which is constitutionally expressed on cardiac fibroblasts and may inhibit the profibrotic cellular activities of cardiac fibroblasts through the modulation of Ca^2+^ homeostasis [64,67,68,108,109,110]. In our previous study, we identified the diverse protein expression in transient receptor potential (TRP) channels, phosphorylated phospholipase C (PLC), and the stromal interaction molecule (STIM)1 between LA and RA fibroblasts [59]. Phospholipase C (PLC), the second messenger of multiple profibrotic cytokines [111,112], hydrolyzes phosphatidylinositol 4,5-bisphosphate (PIP2), thereby producing diacylglycerol (DAG) and inositol trisphosphate (IP3). TRP channels are one of the gateways for extracellular Ca^2+^ entry [113]. TRP channels, which can be activated by DAG [114,115], are upregulated in patients with AF and contribute to atrial fibrosis [116]. The blocking of TRP channels attenuates Ca^2+^ entry-induced collagen production and myofibroblast differentiation in atrial fibroblasts [117]. IP3 signaling activates Ca^2+^ release from the ER [118,119]. The emptying of Ca^2+^ from the ER can be sensed by STIM1, a single-pass membrane protein in the ER membrane, leading to the activation of the store-operated Ca^2+^ entry [120]. In ovariectomized rats, 17β-estradiol-induced atrial natriuretic peptide (ANP) production is higher in the RA than in the LA [64]. This finding of higher estrogen-induced ANP is consistent with findings from other studies, which have revealed that the RA expresses higher ANP protein and gene levels than the LA in various species [17,70,71,72,73,74]. ANP/cGMP signaling can attenuate Ca^2+^ influx by downregulating the phosphorylation of the TRP channels, thereby decreasing profibrotic signaling [75,121]. Interestingly, estrogen receptor-α signaling can also activate cGMP and eNOS and protect against cardiac remodeling [65,66]; hence, this chamber-specific protein diversity in estrogen receptor-α/ANP/cGMP promotes the differences in atria fibrogenesis through NO and Ca^2+^ signaling crosstalk.

In patients with AF, gene expression involved in the Wnt signaling pathway is greater in LA tissue than in RA tissue [87]. The noncanonical Wnt signaling phosphorylates PLC and increases IP3 production, leading to the induction of Ca^2+^ homeostasis [88]. In addition, cGMP attenuates Wnt stimulator-activated Ca^2+^ mobilization [122]. Accordingly, the LA constitutionally expresses a higher amount of the Ca^2+^ activator, whereas RA expresses greater levels of the Ca^2+^ signal inhibitor, leading to the regional diversity in atrial fibrogenesis.

### 3.2. TGF-β Paracrine Effect

TGF-β is the key driving force of atrial fibrosis [123] and has higher levels in patients with AF and atrial remodeling or fibrosis than in patients with sinus rhythm [124,125]. TGF-β1 induces profibrotic cellular activities of atrial fibroblasts through downstream Smad2/3 signaling [126]. Smad6 can be considered as an inhibitory Smad that interferes with the phosphorylation of Smad2/3 [76]. The overexpression of Smad6 decreases the collagen expression of fibroblasts [127], and compared with LA tissue, RA tissue exhibits greater Smad6 gene expression [77]. Bone morphogenetic protein (BMP) is part of the TGF-β signaling superfamily. BMP plays an essential role in the cardiac development and pathogenesis of various cardiovascular diseases [78]. BMP-10, a gene that is 282-fold greater expressed in RA than in LA, reduces the collagen-production capability of cardiac fibroblasts [18,20,128]. Moreover, BMP-10 transgenic mice or mice treated with BMP-10 exhibit lower levels of cardiac fibrosis. Consequently, the RA expresses a greater amount of TGF-β inhibitory factor and induced lower levels of fibrogenesis than the LA. However, the LA exhibits greater activating transcription factor 3, which can induce an increased amount of TGF-β1 and greater collagen type I production in fibroblasts [89,129]. The LA exhibits greater iron deposition [7], and the inhibition of iron deposition suppresses TGF-β production and reduces the extent of myocardial fibrosis [90]. Iron-reduction therapy can decrease the occurrence of paroxysmal AF in patients with chronic iron overload [130]. Patients with iron overload exhibited increased LA stiffness, which is a strong independent predictor of AF [131,132], suggesting that the LA has a higher propensity for TGF-β production and has greater potential for activating greater fibrosis as a result of iron overload.

Adrenomedullin can also decrease collagen production capability by decreasing TGF-β1 production in fibroblasts [62]. ANP/cGMP decreases the profibrotic cellular activities of fibroblasts by interfering with Smad3 signaling [133], suggesting that cross-talk between NO and the anti-TGF-β pathway contributes to the lower fibrosis in the RA. The LA expresses a higher level of Mir-10b and Mir-208 than the RA [20,94]. The upregulation of circulating Mir-10b can predict myocardial fibrosis in patients with hypertrophic cardiomyopathy [134]. The inhibition of Mir-10b can decrease TGF-β1 production and myofibroblast differentiation [91,92], and in patients with dilated cardiomyopathy, Mir-208 is positively correlated with the severity of cardiac fibrosis [135]. The overexpression of Mir-208 increases the expression of endoglin, a coreceptor of TGF-β, and augments the collagen expression of myofibroblasts [95]. By contrast, Mir-135a, which is more highly expressed in the RA than in the LA, can inhibit TGF-β1 production and currents through TRP channels, thereby decreasing the collagen production and myofibroblast differentiation of cardiac fibroblasts [20,82]. These epigenetic diversities in TGF-β modulation may also contribute to atrial regional fibrogenic diversity.

### 3.3. Oxidative Stress Signaling

Oxidative stress plays an essential role in the pathogenesis of AF [136]. Reactive oxygen species (ROS) derived from nicotinamide adenine dinucleotide phosphate oxidase (Nox)-4 potentiates the TGF-β-induced collagen type I transcriptional activity of atrial fibroblasts [137]. ROS can also upregulate the production of TGF-β in cardiac fibroblasts [138]. Furthermore, oxidative stress increases the gene expression of the Ang type I receptor, thereby increasing the responsiveness of cardiac fibroblasts to Ang II [139]. Moreover, oxidative stress mediates the atrial fibrosis and AF vulnerability of diabetic animals [140]; hence, increased production of oxidative stress may enhance atrial fibrogenesis. Knocking out estrogen receptor-α increases Ang II-induced oxidative stress production [69]. Our previous study showed that LA tissue exhibited greater levels of oxidative stress and atrial fibrosis compared to RA in reduced ejection fraction HF rats measured by echocardiography [36]. The RA has a greater expression of heat shock protein 70, which may result in a cytoprotective effect against oxidative stress and, consequently, lower levels of fibrogenesis [79,80]. Hepcidin is an antimicrobial protein that controls iron metabolism in mammals [141]. Hepcidin also decreases oxidative stress in rats with myocardial infarction [81]. The RA expresses greater gene expression of hepcidin than the LA [20]. Adrenomedullin also attenuates oxidative stress production, leading to lower levels of cardiac fibrosis in hypertensive rats [63]. Moreover, Mir-100, which is more highly expressed in the RA than in the LA, decreases ROS production by targeting Nox 4 [83,84]. By contrast, the inhibition of the LA-enriched Mir-10b can decrease oxidative stress production [93]. In summary, these modulators protect RA against oxidative injury, thereby decreasing levels of atrial fibrogenesis.

## 4. Conclusions

**Figure 3:**
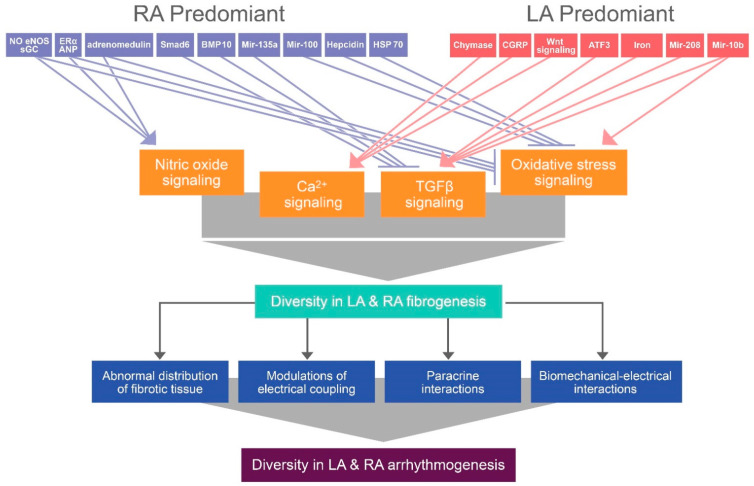
Illustration of the proposed mechanism that contributes to differential atrial fibrogenesis-induced arrhythmogenesis between the LA and RA. Diverse gene or protein expression induces differential atrial fibrogenesis by modifying nitric oxide, Ca^2+^, transforming growth factor (TGF)- β, and oxidative stress signaling, thereby activating diversity in atrial arrhythmogenesis through the abnormal distribution of fibrotic tissue, modulations of electrical coupling, paracrine interactions, and biomechanical–electrical interactions. *ERα*: estrogen receptor-α, *eNOS*: endothelial nitric oxide synthase, *sGC*: soluble guanylyl cyclase, *ANP*: atrial natriuretic peptide, *Smad6*: SMA and MAD-related protein 6, *BMP-10*: bone morphogenetic protein-10, *HSP-70*: heat shock protein-70, *Mir*: micro RNA, *CGRP*: calcitonin gene-related peptide, *ATF3*: activating transcription factor 3.

In conclusion, as summarized in Figure 3, various factors with heterogeneous distribution between the LA and RA may activate diverse atrial regional fibrogenesis through NO, Ca^2+^, TGF-β, and oxidative stress signaling pathways, thereby inducing different atrial electrical remodeling.

## Figures and Tables

**Table 1:** List of targets that activate the atrial regional diversities in fibrogenesis.

Predominant Chamber	Molecules	Signaling That Induces Atrial Diversities	Effects of Molecules on the Signaling	References
RA	Nitric oxide	Nitric oxide signaling	+	[6,61]
	eNOS	Nitric oxide signaling	+	[61]
	sGC	Nitric oxide signaling	+	[61]
	Adrenomedullin	Nitric oxide signaling	+	[18]
		TGF-β signaling	−	[62]
		Oxidative stress signaling	−	[63]
	Estrogen receptor-α	Nitric oxide signaling	+	[64,65,66]
		Ca^2+^ signaling	−	[67,68]
		Oxidative stress signaling	−	[69]
	ANP	Nitric oxide signaling	+	[17,64,70,71,72,73,74]
		Ca^2+^ signaling	−	[75]
	Smad6	TGF-β signaling	−	[76,77]
	BMP-10	TGF-β signaling	−	[18,20,78]
	HSP-70	Oxidative stress signaling	−	[79,80]
	Hepcidin	Oxidative stress signaling	−	[20,81]
	Mir-135a	Ca^2+^ signaling	−	[20,82]
		TGF-β signaling	−	[82]
	Mir-100	Oxidative stress signaling	−	[83,84]
LA	Chymase	Ca^2+^ signaling	+	[85]
	CGRP	Ca^2+^ signaling	+	[86]
	Wnt related gene	Ca^2+^ signaling	+	[87,88]
	ATF3	TGF-β signaling	+	[89]
	Iron	TGF-β signaling	+	[7,90]
	Mir-10b	TGF-β signaling	+	[20,91,92]
		Oxidative stress signaling	+	[93]
	Mir-208	TGF-β signaling	+	[94,95]

eNOS: endothelial nitric oxide synthase, sGC: soluble guanylyl cyclase, ANP: atrial natriuretic peptide, Smad6: SMA and MAD-related protein 6, BMP-10: bone morphogenetic protein-10, HSP-70: heat shock protein-70, Mir: micro RNA, CGRP: calcitonin gene-related peptide, ATF3: activating transcription factor 3.

## References

[1] Kainuma S., Masai T., Yoshitatsu M., Miyagawa S., Yamauchi T., Takeda K., Morii E., Sawa Y. (2011). Advanced left-atrial fibrosis is associated with unsuccessful maze operation for valvular atrial fibrillation. Eur. J. Cardiothorac. Surg..

[2] Oakes R.S., Badger T.J., Kholmovski E.G., Akoum N., Burgon N.S., Fish E.N., Blauer J.J.E., Rao S.N., DiBella E.V.R., Segerson N.M. (2009). Detection and quantification of left atrial structural remodeling with delayed-enhancement magnetic resonance imaging in patients with atrial fibrillation. Circulation.

[3] Ohtani K., Yutani C., Nagata S., Koretsune Y., Hori M., Kamada T. (1995). High prevalence of atrial fibrosis in patients with dilated cardiomyopathy. J. Am. Coll. Cardiol..

[4] Swedberg K., Zannad F., McMurray J.J.V., Krum H., van Veldhuisen D.J., Shi H., Vincent J., Pitt B. (2012). Eplerenone and atrial fibrillation in mild systolic heart failure: Results from the EMPHASIS-HF (Eplerenone in Mild Patients Hospitalization and SurvIval Study in Heart Failure) study. J. Am. Coll. Cardiol..

[5] Xu J., Cui G., Esmailian F., Plunkett M., Marelli D., Ardehali A., Odim J., Laks H., Sen L. (2004). Atrial extracellular matrix remodeling and the maintenance of atrial fibrillation. Circulation.

[6] Park J.H., Lee J.S., Ko Y.G., Lee S.H., Lee B.S., Kang S.M., Chang B.C., Pak H.N. (2014). Histological and biochemical comparisons between right atrium and left atrium in patients with mitral valvular atrial fibrillation. Korean Circ. J..

[7] Swartz M.F., Fink G.W., Lutz C.J., Taffet S.M., Berenfeld O., Vikstrom K.L., Kasprowicz K., Bhatta L., Puskas F., Kalifa J. (2009). Left versus right atrial difference in dominant frequency, K(+) channel transcripts, and fibrosis in patients developing atrial fibrillation after cardiac surgery. Heart Rhythm.

[8] Swartz M.F., Fink G.W., Sarwar M.F., Hicks G.L., Yu Y., Hu R., Lutz C.J., Taffet S.M., Jalife J. (2012). Elevated pre-operative serum peptides for collagen I and III synthesis result in post-surgical atrial fibrillation. J. Am. Coll. Cardiol..

[9] Smorodinova N., Lantová L., Bláha M., Melenovský V., Hanzelka J., Pirk J., Kautzner J., Kučera T. (2015). Bioptic Study of Left and right atrial interstitium in cardiac patients with and without atrial fibrillation: Interatrial but not rhythm-based differences. PLoS ONE.

[10] Marrouche N.F., Wilber D., Hindricks G., Jais P., Akoum N., Marchlinski F., Kholmovski E., Burgon N., Hu N., Mont L. (2014). Association of atrial tissue fibrosis identified by delayed enhancement MRI and atrial fibrillation catheter ablation: The DECAAF study. JAMA.

[11] Akoum N., McGann C., Vergara G., Badger T., Ranjan R., Mahnkopf C., Kholmovski E., Macleod R., Marrouche N. (2012). Atrial fibrosis quantified using late gadolinium enhancement MRI is associated with sinus node dysfunction requiring pacemaker implant. J. Cardiovasc. Electrophysiol..

[12] Skanes A.C., Mandapati R., Berenfeld O., Davidenko J.M., Jalife J. (1998). Spatiotemporal periodicity during atrial fibrillation in the isolated sheep heart. Circulation.

[13] Berenfeld O., Mandapati R., Dixit S., Skanes A.C., Chen J., Mansour M., Jalife J. (2000). Spatially distributed dominant excitation frequencies reveal hidden organization in atrial fibrillation in the Langendorff-perfused sheep heart. J. Cardiovasc. Electrophysiol..

[14] Tai C.T., Lo L.W., Lin Y.J., Chen S.A. (2012). Arrhythmogenic difference between the left and right atria in a canine ventricular pacing-induced heart failure model of atrial fibrillation. Pacing Clin. Electrophysiol..

[15] Rohr S. (2012). Arrhythmogenic Implications of Fibroblast-Myocyte Interactions. Circ. Arrhythm. Electrophysiol..

[16] Pellman J., Zhang J., Sheikh F. (2016). Myocyte-fibroblast communication in cardiac fibrosis and arrhythmias: Mechanisms and model systems. J. Mol. Cell Cardiol..

[17] Chapeau C., Gutkowska J., Schiller P.W., Milne R.W., Thibault G., Garcia R., Genest J., Cantin M. (1985). Localization of immunoreactive synthetic atrial natriuretic factor (ANF) in the heart of various animal species. J. Histochem. Cytochem..

[18] Kahr P.C., Piccini I., Fabritz L., Greber B., Schöler H., Scheld H.H., Hoffmeier A., Brown N.A., Kirchhof P. (2011). Systematic analysis of gene expression differences between left and right atria in different mouse strains and in human atrial tissue. PLoS ONE.

[19] Tabibiazar R., Wagner R.A., Liao A., Quertermous T. (2003). Transcriptional profiling of the heart reveals chamber-specific gene expression patterns. Circ. Res..

[20] Hsu J., Hanna P., Van Wagoner D.R., Barnard J., Serre D., Chung M.K., Smith J.D. (2012). Whole genome expression differences in human left and right atria ascertained by RNA sequencing. Circ. Cardiovasc. Genet..

[21] Spach M.S., Miller W.T., Dolber P.C., Kootsey J.M., Sommer J.R., Mosher C.E. (1982). The functional role of structural complexities in the propagation of depolarization in the atrium of the dog. Cardiac conduction disturbances due to discontinuities of effective axial resistivity. Circ. Res..

[22] Ashihara T., Haraguchi R., Nakazawa K., Namba T., Ikeda T., Nakazawa Y., Ozawa T., Ito M., Horie M., Trayanova N.A. (2012). The role of fibroblasts in complex fractionated electrograms during persistent/permanent atrial fibrillation: Implications for electrogram-based catheter ablation. Circ. Res..

[23] Palacio L.C., Ugarte J.P., Saiz J., Tobón C. (2021). The effects of fibrotic cell type and its density on atrial fibrillation dynamics: An in silico study. Cells.

[24] Tanaka K., Zlochiver S., Vikstrom K.L., Yamazaki M., Moreno J., Klos M., Zaitsev A.V., Vaidyanathan R., Auerbach D.S., Landas S. (2007). Spatial distribution of fibrosis governs fibrillation wave dynamics in the posterior left atrium during heart failure. Circ. Res..

[25] Callegari S., Macchi E., Monaco R., Magnani L., Tafuni A., Croci S., Nicastro M., Garrapa V., Banchini A., Becchi G. (2020). Clinicopathological bird’s-eye view of left atrial myocardial fibrosis in 121 patients with persistent atrial fibrillation: Developing architecture and main cellular players. Circ. Arrhythm. Electrophysiol..

[26] Hsieh M.H., Lin Y.J., Wang H.H., Lo L.W., Chang S.L., Yan Y.L., Chou T.Y., Chen S.A., Yeh H.I. (2013). Functional characterization of atrial electrograms in a pacing-induced heart failure model of atrial fibrillation: Importance of regional atrial connexin40 remodeling. J. Cardiovasc. Electrophysiol..

[27] Poulet C., Künzel S., Büttner E., Lindner D., Westermann D., Ravens U. (2016). Altered physiological functions and ion currents in atrial fibroblasts from patients with chronic atrial fibrillation. Physiol. Rep..

[28] Maleckar M.M., Greenstein J.L., Giles W.R., Trayanova N.A. (2009). Electrotonic coupling between human atrial myocytes and fibroblasts alters myocyte excitability and repolarization. Biophys. J..

[29] Kamkin A., Kiseleva I., Wagner K.D., Lammerich A., Bohm J., Persson P.B., Günther J. (1999). Mechanically induced potentials in fibroblasts from human right atrium. Exp. Physiol..

[30] Zhan H., Xia L. (2013). Excitation-contraction coupling between human atrial myocytes with fibroblasts and stretch activated channel current: A simulation study. Comput Math. Methods Med..

[31] Yoon Y.E., Kim H.J., Kim S.A., Kim S.H., Park J.H., Park K.H., Choi S., Kim M.K., Kim H.S., Cho G.Y. (2012). Left atrial mechanical function and stiffness in patients with paroxysmal atrial fibrillation. J. Cardiovasc. Ultrasound.

[32] Musa H., Kaur K., O’Connell R., Klos M., Guerrero-Serna G., Avula U.M.R., Herron T.J., Kalifa J., Anumonwo J.M.B., Jalife J. (2013). Inhibition of platelet-derived growth factor-AB signaling prevents electromechanical remodeling of adult atrial myocytes that contact myofibroblasts. Heart Rhythm.

[33] Ramos-Mondragón R., Vega A.V., Avila G. (2011). Long-term modulation of Na+ and K+ channels by TGF-β1 in neonatal rat cardiac myocytes. Pflugers Arch.

[34] Verheule S., Sato T., Everett T.t., Engle S.K., Otten D., Rubart-von der Lohe M., Nakajima H.O., Nakajima H., Field L.J., Olgin J.E. (2004). Increased vulnerability to atrial fibrillation in transgenic mice with selective atrial fibrosis caused by overexpression of TGF-beta1. Circ. Res..

[35] Choi E.K., Chang P.C., Lee Y.S., Lin S.F., Zhu W., Maruyama M., Fishbein M.C., Chen Z., Rubart-von der Lohe M., Field L.J. (2012). Triggered firing and atrial fibrillation in transgenic mice with selective atrial fibrosis induced by overexpression of TGF-β1. Circ. J..

[36] Chung C.C., Kao Y.H., Yao C.J., Lin Y.K., Chen Y.J. (2017). A comparison of left and right atrial fibroblasts reveals different collagen production activity and stress-induced mitogen-activated protein kinase signalling in rats. Acta Physiol..

[37] Tao H., Shi K.H., Yang J.J., Huang C., Liu L.P., Li J. (2013). Epigenetic regulation of cardiac fibrosis. Cell Signal..

[38] Bhatia H., Verma G., Datta M. (2014). miR-107 orchestrates ER stress induction and lipid accumulation by post-transcriptional regulation of fatty acid synthase in hepatocytes. Biochim. Biophys. Acta.

[39] Hsieh W.J., Lin F.M., Huang H.D., Wang H. (2014). Investigating microRNA-target interaction-supported tissues in human cancer tissues based on miRNA and target gene expression profiling. PLoS ONE.

[40] Li S., Gao Y., Liu Y., Li J., Yang X., Hu R., Liu J., Zhang Y., Zuo K., Li K. (2020). Myofibroblast-derived exosomes contribute to development of a susceptible substrate for atrial fibrillation. Cardiology.

[41] Zhou Y., Xu W., Han R., Zhou J., Pan Z., Rong H., Li J., Xu C., Qiao G., Lu Y. (2012). Matrine inhibits pacing induced atrial fibrillation by modulating I(KM3) and I(Ca-L). Int. J. Biol Sci..

[42] Karam B.S., Chavez-Moreno A., Koh W., Akar J.G., Akar F.G. (2017). Oxidative stress and inflammation as central mediators of atrial fibrillation in obesity and diabetes. Cardiovasc. Diabetol..

[43] Yang Y., Zhao J., Qiu J., Li J., Liang X., Zhang Z., Zhang X., Fu H., Korantzopoulos P., Letsas K.P. (2018). Xanthine oxidase inhibitor allopurinol prevents oxidative stress-mediated atrial remodeling in alloxan-induced diabetes mellitus rabbits. J. Am. Heart Assoc..

[44] Cai B., Pan Z., Liu Y., Chen N., Lu Y. (2011). Arrhythmogenic potential of oxidative stress in atrial myocytes. Int. J. Cardiol..

[45] Tsai C.T., Tseng C.D., Hwang J.J., Wu C.K., Yu C.C., Wang Y.C., Chen W.P., Lai L.P., Chiang F.T., Lin J.L. (2011). Tachycardia of atrial myocytes induces collagen expression in atrial fibroblasts through transforming growth factor β1. Cardiovasc. Res..

[46] Moreira L.M., Takawale A., Hulsurkar M., Menassa D.A., Antanaviciute A., Lahiri S.K., Mehta N., Evans N., Psarros C., Robinson P. (2020). Paracrine signalling by cardiac calcitonin controls atrial fibrogenesis and arrhythmia. Nature.

[47] Zheng Y., Xia Y., Carlson J., Kongstad O., Yuan S. (2017). Atrial average conduction velocity in patients with and without paroxysmal atrial fibrillation. Clin. Physiol. Funct Imaging.

[48] Nitta T., Ishii Y., Miyagi Y., Ohmori H., Sakamoto S., Tanaka S. (2004). Concurrent multiple left atrial focal activations with fibrillatory conduction and right atrial focal or reentrant activation as the mechanism in atrial fibrillation. J. Thorac Cardiovasc. Surg..

[49] Sanders P., Berenfeld O., Hocini M., Jaïs P., Vaidyanathan R., Hsu L.F., Garrigue S., Takahashi Y., Rotter M., Sacher F. (2005). Spectral analysis identifies sites of high-frequency activity maintaining atrial fibrillation in humans. Circulation.

[50] Berenfeld O. (2007). Quantifying activation frequency in atrial fibrillation to establish underlying mechanisms and ablation guidance. Heart Rhythm.

[51] Sarmast F., Kolli A., Zaitsev A., Parisian K., Dhamoon A.S., Guha P.K., Warren M., Anumonwo J.M., Taffet S.M., Berenfeld O. (2003). Cholinergic atrial fibrillation: I(K,ACh) gradients determine unequal left/right atrial frequencies and rotor dynamics. Cardiovasc. Res..

[52] Jeyaratnam J., Umapathy K., Masse S., Nair K., Farid T., Krishnan S., Nanthakumar K. Relating spatial heterogeneities to rotor formation in studying human ventricular fibrillation. Proceedings of the 2011 Annual International Conference of the IEEE Engineering in Medicine and Biology Societ.

[53] Nakamura T., Kiuchi K., Fukuzawa K., Takami M., Watanabe Y., Izawa Y., Suehiro H., Akita T., Takemoto M., Sakai J. (2021). Late-gadolinium enhancement properties associated with atrial fibrillation rotors in patients with persistent atrial fibrillation. J. Cardiovasc. Electrophysiol..

[54] Lin T., Rillig A., Bucur T., Metzner A., Mathew S., Wissner E., Wohlmuth P., Kuck K.H., Ouyang F., Tilz R.R. (2015). Focal impulse and rotor modulation using the novel 64-electrode basket catheter: Electrogram characteristics of human rotors. Europace.

[55] Morillo C.A., Klein G.J., Jones D.L., Guiraudon C.M. (1995). Chronic rapid atrial pacing. Structural, functional, and electrophysiological characteristics of a new model of sustained atrial fibrillation. Circulation.

[56] Mansour M., Mandapati R., Berenfeld O., Chen J., Samie F.H., Jalife J. (2001). Left-to-right gradient of atrial frequencies during acute atrial fibrillation in the isolated sheep heart. Circulation.

[57] Li B., Luo F., Luo X., Li B., Qi L., Zhang D., Tang Y. (2019). Effects of atrial fibrosis induced by mitral regurgitation on atrial electrophysiology and susceptibility to atrial fibrillation in pigs. Cardiovasc. Pathol..

[58] Ali S.R., Ranjbarvaziri S., Talkhabi M., Zhao P., Subat A., Hojjat A., Kamran P., Müller A.M.S., Volz K.S., Tang Z. (2014). Developmental heterogeneity of cardiac fibroblasts does not predict pathological proliferation and activation. Circ. Res..

[59] Chung C.C., Lin Y.K., Chen Y.C., Kao Y.H., Yeh Y.H., Chen Y.J. (2021). Calcium regulation on the atrial regional difference of collagen production activity in atrial fibrogenesis. Biomedicines.

[60] Chung C.C., Lin Y.K., Chen Y.C., Kao Y.H., Yeh Y.H., Chen Y.J. (2018). Factor Xa inhibition by rivaroxaban regulates fibrogenesis in human atrial fibroblasts with modulation of nitric oxide synthesis and calcium homeostasis. J. Mol. Cell Cardiol..

[61] Brahmajothi M.V., Campbell D.L. (2007). Heterogeneous expression of NO-activated soluble guanylyl cyclase in mammalian heart: Implications for NO- and redox-mediated indirect versus direct regulation of cardiac ion channel function. Channels.

[62] Hao S.L., Yu Z.H., Qi B.S., Luo J.Z., Wang W.P. (2011). The antifibrosis effect of adrenomedullin in human lung fibroblasts. Exp. Lung Res..

[63] Rahman M., Nishiyama A., Guo P., Nagai Y., Zhang G.X., Fujisawa Y., Fan Y.Y., Kimura S., Hosomi N., Omori K. (2006). Effects of adrenomedullin on cardiac oxidative stress and collagen accumulation in aldosterone-dependent malignant hypertensive rats. J. Pharmacol. Exp. Ther..

[64] Jankowski M., Rachelska G., Donghao W., McCann S.M., Gutkowska J. (2001). Estrogen receptors activate atrial natriuretic peptide in the rat heart. Proc. Natl. Acad. Sci. USA.

[65] Fukuma N., Takimoto E., Ueda K., Liu P., Tajima M., Otsu Y., Kariya T., Harada M., Toko H., Koga K. (2020). Estrogen receptor-α non-nuclear signaling confers cardioprotection and is essential to cGMP-PDE5 inhibition efficacy. JACC Basic Transl. Sci..

[66] Hohmann N., Xia N., Steinkamp-Fenske K., Förstermann U., Li H. (2016). Estrogen receptor signaling and the PI3K/Akt pathway are involved in betulinic acid-induced eNOS activation. Molecules.

[67] Thor D., Zhang R., Anderson L., Bose D.D., Dubé G.P., Rahimian R. (2010). Effects of 17 β-estradiol on lipopolysacharride-induced intracellular adhesion molecule-1 mRNA expression and Ca²^+^ homeostasis alteration in human endothelial cells. Vascul Pharmacol..

[68] Sribnick E.A., Del Re A.M., Ray S.K., Woodward J.J., Banik N.L. (2009). Estrogen attenuates glutamate-induced cell death by inhibiting Ca^2+^ influx through L-type voltage-gated Ca^2+^ channels. Brain Res..

[69] Guivarc’h E., Favre J., Guihot A.L., Vessières E., Grimaud L., Proux C., Rivron J., Barbelivien A., Fassot C., Briet M. (2020). Nuclear activation function 2 estrogen receptor α attenuates arterial and renal alterations due to aging and hypertension in female mice. J. Am. Heart Assoc..

[70] Gutkowska J., Thibault G., Januszewicz P., Cantin M., Genest J. (1984). Direct radioimmunoassay of atrial natriuretic factor. Biochem. Biophys. Res. Commun..

[71] Tsunoda K., Hodsman G.P., Sumithran E., Johnston C.I. (1986). Atrial natriuretic peptide in chronic heart failure in the rat: A correlation with ventricular dysfunction. Circ. Res..

[72] Wilcox J.N., Augustine A., Goeddel D.V., Lowe D.G. (1991). Differential regional expression of three natriuretic peptide receptor genes within primate tissues. Mol. Cell Biol..

[73] Onuoha G.N., Alpar E.K., Nicholls D.P., Buchanan K.D. (1999). Calcitonin gene-related peptide, neuropeptide Y and atrial natriuretic peptide distribution in guinea pig heart from paraffin wax-embedded and formalin-cryoprotected tissues. Histochem. J..

[74] Osman A.H., Yuge S., Hyodo S., Sato S., Maeda S., Marie H., Caceci T., Birukawa N., Urano A., Naruse K. (2004). Molecular identification and immunohistochemical localization of atrial natriuretic peptide in the heart of the dromedary camel (Camelus dromedarius). Comp. Biochem. Physiol. A Mol. Integr. Physiol..

[75] Chen W., Oberwinkler H., Werner F., Gaßner B., Nakagawa H., Feil R., Hofmann F., Schlossmann J., Dietrich A., Gudermann T. (2013). Atrial natriuretic peptide-mediated inhibition of microcirculatory endothelial Ca^2+^ and permeability response to histamine involves cGMP-dependent protein kinase I and TRPC6 channels. Arterioscler. Thromb. Vasc. Biol..

[76] Jung S.M., Lee J.H., Park J., Oh Y.S., Lee S.K., Park J.S., Lee Y.S., Kim J.H., Lee J.Y., Bae Y.S. (2013). Smad6 inhibits non-canonical TGF-β1 signalling by recruiting the deubiquitinase A20 to TRAF6. Nat. Commun..

[77] Lin H., Dolmatova E.V., Morley M.P., Lunetta K.L., McManus D.D., Magnani J.W., Margulies K.B., Hakonarson H., del Monte F., Benjamin E.J. (2014). Gene expression and genetic variation in human atria. Heart Rhythm.

[78] Morrell N.W., Bloch D.B., ten Dijke P., Goumans M.J.T.H., Hata A., Smith J., Yu P.B., Bloch K.D. (2016). Targeting BMP signalling in cardiovascular disease and anaemia. Nat. Rev. Cardiol..

[79] Lin Y.K., Lai M.S., Chen Y.C., Cheng C.C., Huang J.H., Chen S.A., Chen Y.J., Lin C.I. (2012). Hypoxia and reoxygenation modulate the arrhythmogenic activity of the pulmonary vein and atrium. Clin. Sci..

[80] Hsiao C.C., Lee C.H., Yang R.C., Chen J.Y., Su T.C., Chang Y.J., Lin C.Y., Tsai Y.G. (2021). Heat shock protein-70 levels are associated with a state of oxidative damage in the development of bronchopulmonary dysplasia. Front. Pediatr..

[81] Bayraktar A., Erbaş D., Akarca Dizakar S., Göktaş T., Ömeroğlu S., Öz Oyar E. (2020). The effect of hepcidin on cardiac ischemia-reperfusion injury. J. Investig. Surg..

[82] Wu Y., Liu Y., Pan Y., Lu C., Xu H., Wang X., Liu T., Feng K., Tang Y. (2018). MicroRNA-135a inhibits cardiac fibrosis induced by isoproterenol via TRPM7 channel. Biomed. Pharmacother..

[83] Cooley N., Cowley M.J., Lin R.C., Marasco S., Wong C., Kaye D.M., Dart A.M., Woodcock E.A. (2012). Influence of atrial fibrillation on microRNA expression profiles in left and right atria from patients with valvular heart disease. Physiol. Genomics..

[84] Li X., Wang Y., Cai Z., Zhou Q., Li L., Fu P. (2021). Exosomes from human umbilical cord mesenchymal stem cells inhibit ROS production and cell apoptosis in human articular chondrocytes via the miR-100-5p/NOX4 axis. Cell Biol. Int..

[85] Wang H., Varagic J., Nagata S., Kon N.D., Ahmad S., VonCannon J.L., Wright K.N., Sun X., Deal D., Groban L. (2020). Differential expression of the angiotensin-(1-12)/chymase axis in human atrial tissue. J. Surg. Res..

[86] Chang Y., Stover S.R., Hoover D.B. (2001). Regional localization and abundance of calcitonin gene-related peptide receptors in guinea pig heart. J. Mol. Cell Cardiol..

[87] Thomas A.M., Cabrera C.P., Finlay M., Lall K., Nobles M., Schilling R.J., Wood K., Mein C.A., Barnes M.R., Munroe P.B. (2019). Differentially expressed genes for atrial fibrillation identified by RNA sequencing from paired human left and right atrial appendages. Physiol. Genomics.

[88] Zhang J., Chandrasekaran G., Li W., Kim D.Y., Jeong I.Y., Lee S.H., Liang T., Bae J.Y., Choi I., Kang H. (2020). Wnt-PLC-IP3-Connexin-Ca^2+^ axis maintains ependymal motile cilia in zebrafish spinal cord. Nat. Commun..

[89] Hasin T., Elhanani O., Abassi Z., Hai T., Aronheim A. (2011). Angiotensin II signaling up-regulates the immediate early transcription factor ATF3 in the left but not the right atrium. Basic Res. Cardiol..

[90] Saito K., Ishizaka N., Aizawa T., Sata M., Iso-o N., Noiri E., Mori I., Ohno M., Nagai R. (2005). Iron chelation and a free radical scavenger suppress angiotensin II-induced upregulation of TGF-beta1 in the heart. Am. J. Physiol Heart Circ. Physiol..

[91] Yan T., Wang X., Wei G., Li H., Hao L., Liu Y., Yu X., Zhu W., Liu P., Zhu Y. (2021). Exosomal miR-10b-5p mediates cell communication of gastric cancer cells and fibroblasts and facilitates cell proliferation. J. Cancer.

[92] Fang C.Y., Yu C.C., Liao Y.W., Hsieh P.L., Ohiro Y., Chu P.M., Huang Y.C., Yu C.H., Tsai L.L. (2020). miR-10b regulated by Twist maintains myofibroblasts activities in oral submucous fibrosis. J. Formos Med. Assoc..

[93] Ruan Z., Li Y., He R., Li X. (2021). Inhibition of microRNA-10b-5p up-regulates HOXD10 to attenuate Alzheimer’s disease in rats via the Rho/ROCK signalling pathway. J. Drug Target..

[94] Novak J., Sana J., Stracina T., Novakova M., Slaby O. (2017). Doxorubicin and liposomal doxorubicin differentially affect expression of miR-208a and let-7g in rat ventricles and atria. Cardiovasc. Toxicol..

[95] Shyu K.G., Wang B.W., Wu G.J., Lin C.M., Chang H. (2013). Mechanical stretch via transforming growth factor-β1 activates microRNA208a to regulate endoglin expression in cultured rat cardiac myoblasts. Eur. J. Heart Fail..

[96] Kazakov A., Hall R., Jagoda P., Bachelier K., Müller-Best P., Semenov A., Lammert F., Böhm M., Laufs U. (2013). Inhibition of endothelial nitric oxide synthase induces and enhances myocardial fibrosis. Cardiovasc. Res..

[97] Wang D., Yu X., Brecher P. (1998). Nitric oxide and N-acetylcysteine inhibit the activation of mitogen-activated protein kinases by angiotensin II in rat cardiac fibroblasts. J. Biol. Chem..

[98] Mookerjee I., Hewitson T.D., Halls M.L., Summers R.J., Mathai M.L., Bathgate R.A.D., Tregear G.W., Samuel C.S. (2009). Relaxin inhibits renal myofibroblast differentiation via RXFP1, the nitric oxide pathway, and Smad2. FASEB J..

[99] Zhang Y.Q., Jiang Y.F., Hong L., Yang H.J., Zhang J.Y., Zhou Y.F. (2019). Role of Endothelial nitric oxide synthase polymorphisms in atrial fibrillation: A PRISMA-compliant meta-analysis. Med. Sci. Monit..

[100] Hamid S.A., Totzeck M., Drexhage C., Thompson I., Fowkes R.C., Rassaf T., Baxter G.F. (2010). Nitric oxide/cGMP signalling mediates the cardioprotective action of adrenomedullin in reperfused myocardium. Basic Res. Cardiol..

[101] Zhang B., Jiang J., Yue Z., Liu S., Ma Y., Yu N., Gao Y., Sun S., Chen S., Liu P. (2016). Store-operated Ca^2+^ entry (SOCE) contributes to angiotensin II-induced cardiac fibrosis in cardiac fibroblasts. J. Pharmacol. Sci..

[102] Yang S., Huang X.Y. (2005). Ca^2+^ influx through L-type Ca^2+^ channels controls the trailing tail contraction in growth factor-induced fibroblast cell migration. J. Biol. Chem..

[103] Ikeda K., Nakajima T., Yamamoto Y., Takano N., Tanaka T., Kikuchi H., Oguri G., Morita T., Nakamura F., Komuro I. (2013). Roles of transient receptor potential canonical (TRPC) channels and reverse-mode Na^+^/Ca^2+^ exchanger on cell proliferation in human cardiac fibroblasts: Effects of transforming growth factor β1. Cell Calcium..

[104] Murata N., Ito S., Furuya K., Takahara N., Naruse K., Aso H., Kondo M., Sokabe M., Hasegawa Y. (2014). Ca^2+^ influx and ATP release mediated by mechanical stretch in human lung fibroblasts. Biochem. Biophys. Res. Commun..

[105] Lang Y.D., Chang S.F., Wang L.F., Chen C.M. (2010). Chymase mediates paraquat-induced collagen production in human lung fibroblasts. Toxicol. Lett..

[106] Saito K., Muto T., Tomimori Y., Maruoka H., Tanaka T., Fukuda Y. (2003). Human chymase stimulates Ca^2+^ signaling in human polymorphonuclear cells. Immunol. Lett..

[107] Al-Rubaiee M., Gangula P.R., Millis R.M., Walker R.K., Umoh N.A., Cousins V.M., Jeffress M.A., Haddad G.E. (2013). Inotropic and lusitropic effects of calcitonin gene-related peptide in the heart. Am. J. Physiol Heart Circ. Physiol..

[108] Grohé C., Kahlert S., Löbbert K., Stimpel M., Karas R.H., Vetter H., Neyses L. (1997). Cardiac myocytes and fibroblasts contain functional estrogen receptors. FEBS Lett..

[109] Watanabe T., Akishita M., He H., Miyahara Y., Nagano K., Nakaoka T., Yamashita N., Kozaki K., Ouchi Y. (2003). 17 beta-estradiol inhibits cardiac fibroblast growth through both subtypes of estrogen receptor. Biochem. Biophys. Res. Commun..

[110] Dworatzek E., Mahmoodzadeh S., Schriever C., Kusumoto K., Kramer L., Santos G., Fliegner D., Leung Y.-K., Ho S.M., Zimmermann W.H. (2019). Sex-specific regulation of collagen I and III expression by 17β-Estradiol in cardiac fibroblasts: Role of estrogen receptors. Cardiovasc. Res..

[111] Kucich U., Rosenbloom J.C., Shen G., Abrams W.R., Hamilton A.D., Sebti S.M., Rosenbloom J. (2000). TGF-beta1 stimulation of fibronectin transcription in cultured human lung fibroblasts requires active geranylgeranyl transferase I, phosphatidylcholine-specific phospholipase C, protein kinase C-delta, and p38, but not erk1/erk2. Arch. Biochem. Biophys..

[112] Mukherjee S., Duan F., Kolb M.R., Janssen L.J. (2013). Platelet derived growth factor-evoked Ca^2+^ wave and matrix gene expression through phospholipase C in human pulmonary fibroblast. Int. J. Biochem. Cell Biol..

[113] Berridge M.J., Bootman M.D., Lipp P. (1998). Calcium--a life and death signal. Nature.

[114] Wedel B., Boyles R.R., Putney J.W., Bird G.S. (2007). Role of the store-operated calcium entry proteins Stim1 and Orai1 in muscarinic cholinergic receptor-stimulated calcium oscillations in human embryonic kidney cells. J. Physiol..

[115] Hofmann T., Obukhov A.G., Schaefer M., Harteneck C., Gudermann T., Schultz G. (1999). Direct activation of human TRPC6 and TRPC3 channels by diacylglycerol. Nature.

[116] Zhang Y.-J., Ma N., Su F., Liu H., Mei J. (2015). Increased TRPM6 expression in atrial fibrillation patients contribute to atrial fibrosis. Exp. Mol. Pathol..

[117] Harada M., Luo X., Qi X.Y., Tadevosyan A., Maguy A., Ordog B., Ledoux J., Kato T., Naud P., Voigt N. (2012). Transient receptor potential canonical-3 channel-dependent fibroblast regulation in atrial fibrillation. Circulation.

[118] Essen L.O., Perisic O., Katan M., Wu Y., Roberts M.F., Williams R.L. (1997). Structural mapping of the catalytic mechanism for a mammalian phosphoinositide-specific phospholipase C. Biochemistry.

[119] Moccia F., Dragoni S., Lodola F., Bonetti E., Bottino C., Guerra G., Laforenza U., Rosti V., Tanzi F. (2012). Store-dependent Ca(2+) entry in endothelial progenitor cells as a perspective tool to enhance cell-based therapy and adverse tumour vascularization. Curr. Med. Chem..

[120] Stathopulos P.B., Zheng L., Li G.Y., Plevin M.J., Ikura M. (2008). Structural and mechanistic insights into STIM1-mediated initiation of store-operated calcium entry. Cell.

[121] De Mello W.C. (1998). Atrial natriuretic factor reduces cell coupling in the failing heart, an effect mediated by cyclic GMP. J. Cardiovasc. Pharmacol..

[122] Ma L., Wang H.Y. (2006). Suppression of cyclic GMP-dependent protein kinase is essential to the Wnt/cGMP/Ca2+ pathway. J. Biol Chem..

[123] Everett T.H.T., Olgin J.E. (2007). Atrial fibrosis and the mechanisms of atrial fibrillation. Heart Rhythm.

[124] Lin X., Wu N., Shi Y., Wang S., Tan K., Shen Y., Dai H., Zhong J. (2015). Association between transforming growth factor β1 and atrial fibrillation in essential hypertensive patients. Clin. Exp. Hypertens.

[125] Sun Y., Huang Z.Y., Wang Z.H., Li C.P., Meng X.L., Zhang Y.J., Su F., Ma N. (2015). TGF-β1 and TIMP-4 regulate atrial fibrosis in atrial fibrillation secondary to rheumatic heart disease. Mol. Cell Biochem..

[126] Du L., Qin M., Yi Y., Chen X., Jiang W., Zhou L., Zhang D., Xu K., Yang Y., Li C. (2017). Eplerenone prevents atrial fibrosis via the TGF-β signaling pathway. Cardiology.

[127] Seet L.F., Toh L.Z., Finger S.N., Chu S.W., Stefanovic B., Wong T.T. (2016). Valproic acid suppresses collagen by selective regulation of Smads in conjunctival fibrosis. J. Mol. Med..

[128] Qu X., Liu Y., Cao D., Chen J., Liu Z., Ji H., Chen Y., Zhang W., Zhu P., Xiao D. (2019). BMP10 preserves cardiac function through its dual activation of SMAD-mediated and STAT3-mediated pathways. J. Biol. Chem..

[129] Wang X.M., Liu X.M., Wang Y., Chen Z.Y. (2021). Activating transcription factor 3 (ATF3) regulates cell growth, apoptosis, invasion and collagen synthesis in keloid fibroblast through transforming growth factor beta (TGF-beta)/SMAD signaling pathway. Bioengineered.

[130] Zacharski L.R., McKernan L., Metzger M.E., Malone M.G., Samnotra V., Bhargava A., Steiner P.R., Rauwerdink C.A., Ornstein D.L., Cornell C.J. (2010). Remission of paroxysmal atrial fibrillation with iron reduction in haemophilia A. Haemophilia.

[131] Saad A.K., Aladio J.M., Yamasato F., Volberg V.I., Gonzalez Ballerga E., Sordá J.A., Daruich J., Perez de la Hoz R.A. (2021). Analysis of the left atrial function using two-dimensional strain in patients with recent diagnosis of hereditary hemochromatosis. Curr. Probl. Cardiol..

[132] Correia E.T.O., Barbetta L., Silva O., Mesquita E.T. (2019). Left atrial stiffness: A predictor of atrial fibrillation recurrence after radiofrequency catheter ablation—A systematic review and meta-aAnalysis. Arq Bras Cardiol..

[133] Li P., Wang D., Lucas J., Oparil S., Xing D., Cao X., Novak L., Renfrow M.B., Chen Y.F. (2008). Atrial natriuretic peptide inhibits transforming growth factor beta-induced Smad signaling and myofibroblast transformation in mouse cardiac fibroblasts. Circ. Res..

[134] Fang L., Ellims A.H., Moore X.L., White D.A., Taylor A.J., Chin-Dusting J., Dart A.M. (2015). Circulating microRNAs as biomarkers for diffuse myocardial fibrosis in patients with hypertrophic cardiomyopathy. J. Transl. Med..

[135] Satoh M., Minami Y., Takahashi Y., Tabuchi T., Nakamura M. (2010). Expression of microRNA-208 is associated with adverse clinical outcomes in human dilated cardiomyopathy. J. Card Fail..

[136] Samman Tahhan A., Sandesara P.B., Hayek S.S., Alkhoder A., Chivukula K., Hammadah M., Mohamed-Kelli H., O’Neal W.T., Topel M., Ghasemzadeh N. (2017). Association between oxidative stress and atrial fibrillation. Heart Rhythm.

[137] Yeh Y.H., Kuo C.T., Chang G.J., Qi X.Y., Nattel S., Chen W.J. (2013). Nicotinamide adenine dinucleotide phosphate oxidase 4 mediates the differential responsiveness of atrial versus ventricular fibroblasts to transforming growth factor-β. Circ. Arrhythmia Electrophysiol..

[138] Li P.F., Dietz R., von Harsdorf R. (1999). Superoxide induces apoptosis in cardiomyocytes, but proliferation and expression of transforming growth factor-β1 in cardiac fibroblasts. FEBS Lett..

[139] Anupama V., George M., Dhanesh S.B., Chandran A., James J., Shivakumar K. (2016). Molecular mechanisms in H_2_O_2_-induced increase in AT1 receptor gene expression in cardiac fibroblasts: A role for endogenously generated Angiotensin II. J. Mol. Cell Cardiol..

[140] Zhou L., Liu Y., Wang Z., Liu D., Xie B., Zhang Y., Yuan M., Tse G., Li G., Xu G. (2021). Activation of NADPH oxidase mediates mitochondrial oxidative stress and atrial remodeling in diabetic rabbits. Life Sci..

[141] Ganz T. (2003). Hepcidin, a key regulator of iron metabolism and mediator of anemia of inflammation. Blood.

